# Cultural hegemony? Educators’ perspectives on facilitating cross-cultural dialogue

**DOI:** 10.3402/meo.v21.33145

**Published:** 2016-11-25

**Authors:** Zareen Zaidi, Daniëlle Verstegen, Rashmi Vyas, Omayma Hamed, Tim Dornan, Page Morahan

**Affiliations:** 1 Division of General Internal Medicine, Department of Medicine, University of Florida, Gainesville, FL, USA; 2 Department of Educational Development and Research, Faculty of Health, Medicine and Life Sciences, Maastricht University, Maastricht, The Netherlands; 3 FAIMER (Foundation for Advancement of International Medical Education and Research), Philadelphia, PA, USA; 4 Quality & Academic Accreditation Unit, Medical Education Department, Faculty of Medicine, King Abdulaziz University (KSA), Jeddah, Saudi Arabia; 5 Cairo University, Egypt; 6 FAIMER Institute, Drexel University College of Medicine, Philadelphia, PA, USA

**Keywords:** cross-cultural communication, educational cultural hegemony, culturally responsive andragogy, critical consciousness, discourse analysis

## Abstract

**Background:**

We live in an age when education is being internationalized. This can confront students with ‘cultural hegemony’ that can result from the unequal distribution of power and privilege in global society. The name that is given to awareness of social inequality is ‘critical consciousness’. Cross-cultural dialogue provides an opportunity for learners to develop critical consciousness to counter cultural hegemony. The purpose of this research was to understand how learners engage with cross-cultural dialogue, so we can help them do so more effectively in the future.

**Method:**

The setting for this research was an online discussion in an international health professions educator fellowship program. We introduced scenarios with cultural references to study the reaction of participants to cultural conversation cues. We used an inductive thematic analysis to explore power and hegemony issues.

**Results:**

Participants reflected that personally they were more likely to take part in cross-cultural discussions if they recognized the context discussed or had prior exposure to educational settings with cultural diversity. They identified barriers as lack of skills in facilitating cross-cultural discussions and fear of offending others. They suggested deliberately introducing cultural issues throughout the curriculum.

**Conclusion:**

Our results indicate that developing critical consciousness and cross-cultural competency will require instructional design to identify longitudinal opportunities to bring up cross-cultural issues, and training facilitators to foster cross-cultural discussions by asking clarifying questions and navigating crucial/sensitive conversations.

‘No culture ever developed, bloomed and matured without feeding on other cultures … reciprocal influences and intermingling’

Maria Vargas Llosa 2010 Nobel Prize in Literature.

Global communication and internationalization are now integral parts of many higher education programs (1). Participants are most likely to develop the mutual trust, on which the long-term success of such programs depends, when they are ‘culturally competent’ (2–4). In the absence of trust, learners from minority backgrounds have reported a variety of emotional and physical symptoms (5). Cultural competence means more than acquiring knowledge, attitude, and skills. Cultural competence is the ability to interact respectfully with colleagues from any culture and requires ‘critical consciousness’ (6). The philosopher Paulo Freire described critical consciousness as an in-depth reflective understanding of the world, which takes account of social relationships and power dynamics (7, 8).

Acquiring such critical consciousness and reflective ability is not easy, because not every member of society has equal access to money, status, knowledge, information, or even public discourse and communication (9–11). Those with best access may be so influential that they strongly inform what less privileged people perceive as ‘reality’; they enshrine what Gramsci described as the ‘only sensible worldview’ into laws, rules, norms, and habits (12). This results in ‘hegemony’, whereby the most powerful members of society determine what is ‘real’ within a culture. Language, which is an integral part of hegemony, exercises power. Researchers use the word ‘discourse’ to describe ways in which the culturally embedded use of language is a social practice, determined by, and determining, other social practices (13, 14) and how genres and topics of language in public use exercise power (10).

All of this means that those of us, who increasingly educate learners from diverse backgrounds, need to understand which factors promote or hinder cross-cultural dialogue in health professions education (15, 16). Interactions between teachers and learners within classrooms impact engagement of learners and have been shown to replicate cultural hegemony rather than challenge the interactional patterns between dominant and subordinate communities in the wider society (17). In our earlier research with an online educational setting, we wondered if faculty and/or participants experienced ‘educational cultural hegemony’, finding it inappropriate to bring up cultural differences, or if other factors hindered cross-cultural dialogue. We have operationally defined ‘educational cultural hegemony’ as educational practices where teachers assume that the content and task is ‘culture free’ and, therefore, implicitly discourage bringing in personal cultural context. We found that health professions educators from over 40 countries rarely mentioned culture; they focused rather on the specific educational content and tasks (18). As a follow-up to the study, here we aim to explore educational cultural hegemony further by analyzing participant responses to a direct call to discuss cultural issues. The critical theory paradigm (19) with Gramsci's theory of ‘cultural hegemony’ (7, 12) and Freire's ‘critical consciousness’ provided the conceptual framework. Our research questions were: ‘How do participants in an online cross-cultural educational learning setting react to cultural conversation cues?’ and ‘What factors do participants describe that they experienced that hinder or promote readiness to discuss culture in a professional development curriculum?’

## Methods

### Educational setting and participants

Institutional Review Board (IRB) approval was obtained through Foundation University, Pakistan, on commencement of the study. The Foundation for Advancement of International Medical Education & Research (FAIMER) Institute, established in 2001 (20–22), provides a 2-year part time fellowship to develop cohorts of 16 mid-career health professions faculty from Latin America, Africa, the Middle East, and Asia to act as educational research scholars and change agents within a global community of practice (23). During 2013–2014, 164 FAIMER Institute alumni (2001–2011) were invited to convene an online asynchronous discussion on a topic of their choice. The first three authors (FAIMER Institute alumni) convened a 2-week conversation from November 3 to 24, 2014, focusing on participants’ readiness to engage in cultural dialogue and exploring the community's thoughts about ‘educational cultural hegemony’. The participants were current FAIMER Fellows, FAIMER alumni, and faculty. The participants (n = 23) were current FAIMER Fellows (n = 19), FAIMER alumni (n = 3), and faculty (n = 1). This generated conversations and activity in the form of ‘posts’, which were compiled and analyzed for this study.

The FAIMER Institute curriculum includes two (3- and 2-week) residential sessions a year apart in Philadelphia and two 11-month e-learning periods conducted *via* a listserv. During the total immersion residential sessions, Fellows are encouraged to share information about their culture, particularly during structured ‘Learning Circle’ activities (24–26). The listserv is used for formal e-learning modules, alumni-designed community conversations, and as an informal resource network and a social support network for Fellows. During the formal e-learning sessions, Fellows are required to post ‘at least one substantive comment that advances the topic’, but are not given any specific guidelines to deliberately post comments to promote multicultural discourse (18).

### Epistemology and methodology

Epistemology or theory of knowledge guides methodological choices and is considered to be critical or axiological in qualitative research (27). This qualitative research has a subjectivist epistemology and is located in the critical theory paradigm (28), which allows for historical insights and is particularly suitable for analysis of online dialogue such as in this study, which is shaped by social, political, cultural, economic, ethnic, and gender values crystalized over time.

#### Critical reflexivity

‘Critical reflexivity’ allows researchers to explore their own relative positions on the topic. In keeping with critical research practice (28), the researchers were also the facilitators of the discussion and employed critical reflexivity to self-consciously explore their own positions on the data set. The first three authors are FAIMER Fellows who have worked in academic positions in Pakistan and United States (ZZ), India and United States (RV), and Egypt and Saudi Arabia (OH). DV and TD faculty are faculty at the University of Maastricht and work with international students. PM is the founding director of FAIMER. To prevent implicit bias, all researchers, using Skype^®^ calls and emails, explored how their perspectives regarding culture were shaped over the years of interaction with learners from different backgrounds, commented on documents, and helped identify preconceptions that may impact data analysis.

#### Preparation of scenarios

The authors purposefully crafted cultural cues or ‘scenarios’ to provide a rich situation, which allowed the researchers to analyze expressive texts explaining why people found particular components of the scenarios of interest (Table 1). The scenarios were crafted to reflect scenarios that are similar to those the researchers had noted over years of facilitating discussions on multicultural list-serves. While writing the scenarios, researchers discussed among themselves how participants occasionally brought up their culture explicitly in discussions, whereas sometimes the inference to their culture was more subtly embedded in the conversation, and therefore, the scenarios used in the study were written to replicate their own ‘lived experience’ as facilitators. Two (scenarios 1 and 3) had very explicit cultural context and less strong relation to education. Scenario 1, ‘The Dilemma of Yes and No’, described cultural differences in India regarding non-verbal communication through a description of head nodding. Scenario 3, ‘Who Says Islam is Tough’, described the Tanoura Dance, a Sufi custom in Islam, of worship through dance. The other two scenarios (scenarios 2 and 4), ‘Multicultural Learning Environments and Educators’ and ‘Program Evaluation’, had a subtler cultural context embedded in an educational setting that pointed out educational cultural hegemony.

**Table 1:** Description of scenarios

Scenario in brief with research design focus	The scenarios as provided to participants
Scenario 1: ‘The Dilemma of Yes and No’ described cultural differences in non-verbal communication through a description of head nodding horizontally (side to side – more of a circular motion). This is used customarily in South India to say ‘Yes’, while in the United States the same movement means ‘No’.	We recently moved to the United States from South India. My daughter who is studying in elementary school came to me with a question. My daughter said that when teacher asked in the class if she had understood and she said yes by shaking her head the teacher would come to her seat and say ‘don't worry I will help you’. My daughter was puzzled. I of course now enlightened by my Ethiopian friend's experience asked her immediately how did she shake her head. My daughter showed me – horizontally side to side (more of a circular motion) as is customary in South India to say ‘Yes’ which means ‘No’ in United States. I laughed and explained to her that head shaking means different things in different culture. What is considered a ‘Yes’ in South India is a ‘No’ somewhere else and vice versa. So I told her to just speak up and say that she had understood.
Scenario 2: ‘Multicultural Learning Environments and Educators’ described difficulties faced by facilitators in multicultural learning environments. The dilemma presented was how to achieve balance between providing extra support to students who are not accustomed to the critical reflection skill needed as part of the course, and treating all students similarly.	I would agree with you about the difficulty of setting standards when the tests are not as good as they could be, and the teachers are not aware of this problem. However … This view will not help things to improve and there is a way to improve things by using standard setting methods – not necessarily setting the standards (yet). When I was in New Mexico, I worked with Miriam Friedman Ben David for several years in assessment. She and a team of people developed an assessment system for the entire institution. Standard setting was part of it. The exercise of setting standards is highly educational for all teachers who participate. One of the most powerful strategies for changing the quality of tests is when the teachers realize that the tests they have been writing and using are not the best or even OK. They are not interested in someone telling them this. However, when this realization takes place during a standard setting exercise, when teachers examine the questions, one by one, and debate the answers and the assumptions about the questions and the answers it becomes self-evident that the questions are poorly done, ambiguous, that the some of the items are not important, and so on. They realize this in a group of their peers, in a group of ‘experts’ and they see that they don't agree about things and that a standard makes sense. *Standard setting as an educational event* can help to develop the awareness among teachers about poor quality tests that you are suggesting needs to happen and can lead to setting the standards for tests that are better and collectively created by my more aware teachers.
Scenario 3: ‘Who Says Islam is Tough’ described the Tanoura Dance (‘whirling darawish’), a Sufi custom in Islam, of worship through dance aimed at bringing about cleansing and awakening of the heart.	See how Egyptians feel the beauty of God and how they worship HIM through a dance. Tannura Dance (Al Daraweesh) has a very special characteristic as it relies on the dancer's unlimited moves in circles. Believers of this concept see the universe stems from the same point of rotation. As the universe starts and ends from the same point, the dancer will always start and end his movement from that point. He moves anticlockwise, very much like the pilgrims’ movement around Kaba (The Muslims’ holy shrine). The dance is symbolic of our life journey. The dancer brings his colorful skirts (diversity in life) up and down projecting the ups and downs of our lives. The four drums (Duff) in his hands represent the four seasons. We revolve in life. We give birth and new life. We revolve and then ends up subliming into the divine soul to start another life there in the other world. Once the dancer stretches his right hand upward and pointing down with his left hand, he would be establishing the connection between the earth and the sky. Moving in circles, the dancer is very much like alleviating his wordily burdens, reaching ecstasy in a symbolical attempt to approach heaven and sublime into the divine soul. Once he unties the belt around his waist, the dancer would be rhetorically moving upward to heaven. SUFISM is the remembrance of the divine within us. Sufi practice is aimed at bringing about cleansing and awakening of the heart. ‘Dhikir’ of Sufis is the origin of Tannura Dance, which carries its philosophy. Enjoy watching Tannura Dance on this link: http://youtube/yOxiyz1pRSI
Scenario 4: ‘Program Evaluation’ described the impact of cultural uniformity among participants within a leadership program in India, compared to the FAIMER Institute with participants from across the globe.	Why is the CML group (FAIMER Regional Institute, Ludhiana India) more active on the ml-web? I think one of the reasons may be a ‘uniformity’ or similarity of the regional Institute fellows? In contrast, when you look at the Philadelphia crowd, there is a wide variety of fellows. Some may have difficulty with language and expression? Others may not understand the format of certain web discussions or not find it interesting/stimulating. Others may not think the discussion is useful because they cannot relate it to their own work and hence the discussion for them is very abstract and only on paper? I can imagine that a restaurant making soup in Disney World will need to cater to many different taste buds as compared to a small suburban restaurant in a quiet neighborhood. Perhaps some of the fellows involved with the CML regional institute can give some details of their fellows and discussion; I expect that although the topics will be the same but the discussion will be more contextual, to India's medical Education, hence the interest? So coming back to the Philadelphia listserv, one of the ways that we are using to consider stakeholder's needs is to make ml-web (listserv) teams with a wide variety of fellows. Also the planning in Philadelphia and the conference calls help.

#### Design of the on-line conversation and data collection

Three of the authors (ZZ, RV, and OH) facilitated the conversation. They asked participants to pick one of the four scenarios and comment (post) on the scenario or describe why the scenario they had picked resonated with them. The facilitators then asked participants to share their thoughts on whether they felt it is difficult or appropriate to probe culture on a professional list-serve and whether they felt discussing culture on a professional listserv had relevance to health professions education. All posts were compiled into a 63-page document, which was used for data analysis.

#### Analytic procedures

The researchers used Braun and Clarke's framework for theoretical thematic analysis (29). All researchers read the 63-page document. Keeping in mind the research question and following the six phases for thematic analysis described by Braun and Clarke, ZZ and RV, working independently, highlighted material of interest and identified typical stories, patterned responses, and comments. Themes were identified based on the ‘richness’ of comments rather than the percentages of responses (29). All researchers discussed the highlighted material and comments. ZZ maintained an audit trail back to the original data and kept notes about the discussions. She then wrote the narrative of results proceeding from description to interpretation to explanation while asking: ‘What does this theme mean?’ ‘What are the assumptions underpinning it?’ ‘What are the implications of this theme?’ (29). All authors contributed their reflexive reactions to the evolving narrative of results.

## Results

The scenarios elicited participation and reflection from a broad cultural spectrum (14 countries, including Nigeria, Liberia, Cameroon, Ghana, Egypt in Africa and the Middle East, Colombia in Latin America, Turkey, India, Pakistan, Bangladesh, China, Philippines, and Indonesia in Asia, and the United States) and included 23 midcareer Fellows (48% female).

Scenarios 1 and 3, in which the reference to culture was obvious and not directly related to education, elicited the most participation, compared to scenarios 2 and 4 where the reference to culture was more subtle and directly pertained to educational settings. The latent theoretical content analysis revealed two main themes. The first pertained to factors the participants perceived as facilitating or impeding their participation in cross-cultural discussions. The second comprised participants’ points of view regarding educational cultural hegemony.

### Factors determining participation in cross-cultural discussions

In response to the cultural cues, participants stated that when they were asked to pick a scenario to comment on, they tended to pick the one they could relate to at either a personal level or its relation to a cultural norm they had previously read about or heard about. Majority of participants chose to comment on the scenarios with an explicit reference to culture, with only four participants choosing to comment on the scenarios with subtle, implicit reference to culture. When they did comment on the subtle reference scenarios, they did so because they felt they related to the scenario having experienced it in real life. On the contrary, participants familiar with Indian or Chinese culture were likely to pick the ‘dilemma of yes/no’ scenario, because they had actually experienced or understood the cultural nuance. Additionally, those who were more familiar and more exposed to different cultures in the education environment, for example, through the Maastricht University medical education programs (Some alumina fellows were pursuing Masters or PhD degrees at Maastricht University) or as FAIMER alumni faculty, were more comfortable in participating in such discussions.

Participants described their lack of ‘experience in multiculturalism and diversity’ as a major barrier to engaging in cross-cultural dialogue. Others mentioned that they ‘preferred to see what others’ experiences were’ or that ‘silence meant understanding’; most did not provide further explanation for their reticence. Some expressed a ‘fear of putting ideas, belief into correct words’ and ‘fear that a response might open a trail of discussions and posts that might need more response and at the end a confrontation that might cause unnecessary tension’. Others noted pragmatic difficulties in taking part in cultural discussions, such as availability of time and other logistical issues: ‘time constraints are the main reason behind my silence … Work duties, responsibilities and deadlines’. Those who were more familiar and more exposed to different cultures in the education environment, for example, through the Maastricht University medical education programs or as FAIMER alumni faculty, were more comfortable in participating in such discussions.

Analysis of the reflections elicited by the scenario ‘Who says Islam is tough?’ was instructive about why participants responded or did not respond to a cultural cue, or how deeply they reflected. One participant explained that: ‘Though I enjoy Sufi music there was no immediate resonance of a story [in my personal experience] to recollect and share’. Other participants who did comment on this scenario focused on the dancer, without in-depth comments that could have more deeply explored the relevance of dance, music and culture to education. The very deliberately worded title ‘Who says Islam is tough?’ did not draw any comment from participants from other parts of the world regarding the specific position of Islam on music and dance. A non-Muslim participant commented that the fear of offending others might stand in the way: ‘… many times its fear of offending others or intruding in their privacy is what leads to silence’. Another said:

Although I appreciate dancing (and music) as a powerful way of expressing ourselves and a very important part of many or our rituals (and in our region, almost a must in social meetings, with a whole world of meaning), I felt in this topic I should learn from others about Islam.

In contrast, a Muslim participant, who had personal experience with the scenario, felt comfortable in reflecting more deeply. This participant provided an ‘insider’ view stating he/she wished for ‘an opportunity to talk briefly and openly about why I was fasting [in the month of Ramadan, during the on-site session]’ and assumed that other participants felt ‘that they were being obtrusive and invading my privacy … was the reason for their silence’.

### Points of view regarding educational cultural hegemony

After the discussion about the scenarios, the authors presented their concept, based on Gramsci's theory of cultural hegemony, that the current education system promotes ‘cultural educational hegemony’ through an emphasis on content and tasks and expectation for learners to leave their culture at the classroom door. Then, the authors invited participants to provide their views on the appropriateness of deliberately introducing cultural issues in educational settings by explicitly asking learners to share information about their culture, political, economic, or gender-related norms. Some participants had clear views on cultural hegemony and expressed that they felt the current educational system does promote hegemony. One participant vividly described it as a phenomenon similar to ‘Imperial neocolonialism, Hollywoodization, McDonaldization’. Another participant stated that, ‘Yes, current education promotes cultural hegemony … I believe in a curriculum that encourages you to deliberately address culture alongside the task’**.

Several participants described cultural issues that they had experienced that were particularly useful to include in health professions curricula. These included learning about festivals in order to incorporate the dates in the education calendar, learning from visiting students about an Amazonian indigenous tribe's ritualistic use of the ancestral ‘Yage’ (drinking a special beverage with hallucinogenic effects), and learning from co-participant ‘Catholic, Protestant, Muslim, Buddhists about their religious beliefs as part of a “Learning Circle”’. These experiences were rich examples of how including culture in learning contributed to understanding about cultural norms in the context of developing respectful classroom relationships, providing health care, or developing trust-based peer learning groups. One participant integrated these ideas in a post:

In medical education; cross-cultural topics should be considered in curriculum (presented with different ratios in formal, hidden and informal curriculum), teaching/learning and assessment methods, in faculty development programs and in setting the learning environment (including hospital routine and policies).

Others expressed strong opinions about inclusion of cross-cultural discussions in curriculum:

Issues such as culture, mode of learning, customs, and ethics should not be presented separately from other content of FAIMER curriculum. If we do so, they risk becoming de-emphasized as fringe elements or of marginal importance and I believe that we all express cultural and other preferences, for example, gender, age, race, religion, etc. (and … most of this is TOTALLY UNCONSIOUS to us). Thus, we INEVITABLY express these preferences even when we are focused on a task (such as education). My conclusion is that since it is inevitable, that we may as well make the “elephant in the living room” (unconscious preferences) visible, and explicitly deal with them.

Though there was a general consensus regarding the importance of facilitating cross-cultural discussion, such as stating that the ‘more transcultural we are the more easily we can adapt across borders’, participants struggled with how to move towards that goal. Several participants discussed the need for coaching on cross-cultural issues. Participants noted the need for planning regarding the how, when, and where to address cross-cultural issues in the curriculum: ‘Consider what learning outcomes you wish the students to achieve from this cross-cultural learning component’; ‘… The plan is the secret and here it is the curriculum map’ and ‘it is important to design curricula that expose the students to diverse cultures’. Another pointed out:

The FAIMER Philadelphia institute has professionals dealing with multiple cultures. Nevertheless, initiation of successful communication within this cultural diversity needs much more efforts/tools and opportunities from all stakeholders.

Other suggestions included facilitated group discussions or ‘guided exchange in a multicultural setting’ to break down walls to allow frank and respectful discussions, skill development in dealing with diversity in attitudes and behavior, and sessions emphasizing respect for others cultural and religious beliefs. Taken as a whole, participants made a case for careful instructional design to explicitly address skills for cross-cultural interaction and understanding in the curriculum.

## Discussion

### Principal findings and meanings

There are three main findings of our research. First, health professions educators who were part of the international curriculum displayed readiness to engage in cross-cultural dialogue when presented with the explicit cultural cues (scenarios). Second, the participants identified specific facilitating promoters and barriers for effective cross-cultural communication. These, with the perceived needs that participants identified, provided our third major finding – specific guidance for future educational design modifications and skill development of teachers to foster cross-cultural exchange.

A few barriers to engagement in cross-cultural discourse appeared noteworthy. Participants reported a fear of running into misunderstanding or even confrontation when posting a remark about another culture, which resulted in silence of some participants. Other researchers have also noted that social norms may inhibit open disagreement and potential confrontation (30, 31). Thus, some participants indicated that they chose to remain silent during cross-cultural interactions in order to hear from others before attempting to enter the conversation. If participants could not personally relate to a cross-cultural topic, they were less inclined to take part in the discussion; conversely, if the topic was of personal interest or participants had a story to tell, they often contributed to the conversation.

However, while some participants noted that at times they wished for an ‘opportunity to talk’ their cultural nuance, in the wake of silence (or absence of a prompting or facilitation), they did not feel comfortable doing so. For others, ‘silence meant understanding’ and they did not feel the need to say more. This is consistent with research that shows that while Western classrooms with a dominant independent culture may be ‘fearful of silence’ (11, 32), silence in international settings may be a sign of respectful deference for (33, 34) those from Eastern or interdependent cultures (33, 34).

It remains puzzling that, although participants agreed that deliberately intertwining cultural issues into the curriculum is a desirable educational practice, they tended to stay away from the scenarios that did so. It is possible that participants found it easier to join in a discussion that explicitly pertained to culture or that they did not note the subtle and implicit ways in which culture was intertwined in educational settings in two of the scenarios. This pattern could even be interpreted as a sign of educational cultural hegemony itself.

The results point to several concrete educational strategies that educators can use to advance cross-cultural dialogue, thus facilitating the opportunity for the transformational learning recommended in health professions education reform (35). First, participants clearly perceived the need to be trained to introduce and handle sensitive cultural topics, particularly if they were to facilitate such discussions, including when and how to pose clarifying questions to deepen the dialogue and how to navigate crucial/sensitive conversations. Second, they suggested that issues related to cross-cultural competence be embedded within the curriculum rather than being addressed out of context. Several suggested a curriculum map to display how, where, and when cross-cultural topics would be addressed.

### Relationship to other publications

Considerable theory and research have shown that cultural exchanges can disrupt fixed beliefs involved in hegemony and can lead people to revise their positions and reinterpret meaning. This process is essential for transformative learning, which is emphasized in health professions education reform today (36–38). However, cultural hegemony is maintained when there is an unwritten rule to limit dialogue to the content or educational task at hand. The scholar Parker Palmer has labelled this ‘educational objectivism’, which assumes teaching requires a distance between teacher and learner and that the self (subjectivity) should be eliminated; with the newer information on the neuroscience of learning, Palmer urged moving to a more relational epistemology and pedagogy (39). The assumption that discussion should be ‘objective’ and limited to the content or task provides a type of ‘common sense’ or ‘cultural hegemony’ that leads to an absence of cross-cultural exchanges and thus minimizes opportunity for critical reflection and transformative learning (40, 41).

### Limitations and strengths

Strengths of this work included documenting the points of view of health care professionals from many countries who are part of a community of practice (3). The study involved a wide cultural spectrum including 14 countries from three continents. However, the numbers of participants is small limiting generalizability and participation was voluntary; thus, there may have been self-selection of ‘believers’ about the importance of cross-cultural issues. Others may not have participated because they had a different point of view and perhaps were afraid of being viewed as an outsider in the discussion – a counter hegemony (42). It is also possible that participants from countries with authoritarian governments may not actively participate for fear of surveillance and monitoring.

This work also serves as a needs assessment by identifying facilitating factors, barriers to, and suggestions for fostering cross-cultural exchange. This is a first step to developing culturally responsive andragogy. The educational design and process (43) created explicit awareness about cross-cultural concepts such as educational cultural hegemony and communication in a community of practice of health professions educators. In this study, we did not delve into how to achieve this goal – the actual educational design. One of the results of the study was, however, clear appreciation of the importance to integrate cross-cultural competencies in the curriculum.

### Implications for health professional educators

To realize the transformational learning urged by various health professions education reform reports (35), educators need to move beyond content and task-focused curricula to engage and learn from cross-cultural discussions. Implementation will require training faculty and students to handle culturally sensitive topics and curriculum design to explicitly incorporate issues related to cross-cultural issues. This process by itself may be a transformative experience for faculty and students (44).

## Acknowledgement and disclosures

The authors thank Stacey Friedman, Associate Director Evaluation and Planning at FAIMER, for her support. This work was supported by the Gatorade Trust through funds distributed by the Department of Medicine, University of Florida, Gainesville, USA, and by the Medical Education Travelling Fellowship awarded by ASME to the first author.
